# Preparation of Copper Oxide Film at Low Temperature in Basic Conditions on a Copper Substrate

**DOI:** 10.3390/ma18071487

**Published:** 2025-03-26

**Authors:** Francesca Irene Barbaccia, Tilde de Caro, Fulvio Federici, Alessio Mezzi, Lucia Sansone, Michele Giordano, Andrea Macchia

**Affiliations:** 1 Department of Technological Innovation Engineering, International Telematic University Uninettuno, Corso Vittorio Emanuele II 39, 00186 Rome, Italy; 2 Youth in Conservation of Cultural Heritage (YOCOCU APS), Via T. Tasso 108, 00185 Rome, Italy; 3 Institute of Nanostructured Materials, National Research Council (ISMN-CNR), Strada Provinciale 35d, 9, 00010 Rome, Italy; tilde.decaro@cnr.it (T.d.C.); fulvio.federici@cnr.it (F.F.);; 4 Institute for Polymers, Composites, and Biomaterials, National Research Council (IPCB-CNR), Piazzale Enrico Fermi 1, 80055 Portici, Italy

**Keywords:** CuO, copper oxide, tenorite, µ-Raman

## Abstract

Copper is widely used in both its metallic form and as oxide across numerous scientific and industrial domains. The primary copper oxides, cuprite (Cu_2_O) and tenorite (CuO), naturally form on the copper surface and play a crucial role in advanced technologies, such as solar cells, lithium batteries, and sensors. Tenorite is appreciated for its optical properties, stability, low toxicity, and reactivity. While copper oxide thin films are traditionally synthesized through thermal treatments and oxidation in alkaline environments, these conventional high-temperature methods not only require significant energy consumption but can also compromise the metal–film interface. This study aims to develop a sustainable alternative approach for forming a homogeneous CuO layer on a copper substrate through environmentally friendly treatments based on low temperature, cost-effective, and time-saving procedures. Three different eco-conscious treatments were investigated: (i) immersion in NaOH basic solution, (ii) exposure to NaOH basic solution vapours, and (iii) a combined treatment involving both immersion and vapour exposure. This green synthesis approach significantly reduces energy consumption compared to traditional thermal methods while maintaining product quality. The surface oxide layer was investigated through an optical microscope (OM), scanning electron microscopy (SEM), spectrocolorimetric analysis, peel-off test, µ-Raman and X-ray photoelectron spectroscopy (XPS) analysis to investigate the surface oxidation state.

## 1. Introduction

Copper is used in many sectors both as an alloy and in the form of oxide. The applications of this metal extend from artistic production [1,2,3] to the medical field by exploiting the antibacterial properties of its oxides [4,5,6], in the industrial sectors as humidity sensors, gas sensors and biosensors [7,8,9], as well as in electronic and thermal application [10,11,12,13]. Additionally, copper is employed in the production of pipelines, wire, sheet, and marine industry components for communication technologies and water purification or desalination systems [14,15,16,17,18].

Copper oxides exist in two primary forms: cuprite Cu_2_O (copper I) or cuprous oxide and tenorite CuO (copper II) or cupric oxide. In addition to these, a third copper oxide, paramelaconite Cu_4_O_3_, has been identified as an intermediate phase between these two structures [19,20,21]. Copper oxide formation is divided into two steps: the initial ionization of copper (Equation (1)), followed by its oxidation (Equations (2) and (3)):

Cu → Cu^n+^ + ne^−^ (1)

Cu^+^ + O^2−^ → Cu_2_O (2)

Cu^2+^ + O^2−^ → CuO (3)

Due to their semiconductive properties, copper oxides are used in the production of solar cells, photo-electro-chemical cells, photocatalysts, lithium-ion batteries, gas sensors, magnetic media, optical devices and thermal control coating in spacecraft [22,23,24]. Cu_2_O has a band gap range of 1.9 and 2.3 eV [20,25,26] whereas CuO, with a band gap of 1.1 and 1.5 eV, exhibits strong sunlight absorption properties, making it a suitable material for energy applications [12,24,27,28].

However, conventional CuO synthesis methods require high-temperature processing, which can induce structural stress at the metal–film interface and compromise film homogeneity [29,30,31]. Additionally, some techniques involve chemical environments that pose environmental and safety risks. For example, chemical vapour deposition (CVD) and physical vapour deposition (PVD) typically operate at high temperatures, leading to excessive energy consumption and potential thermal deformation of the metallic substrate. Moreover, some synthesis techniques involve volatile organic solvents or toxic reagents, increasing environmental and operational hazards [32,33,34]. The use of organometallic precursors in CVD, for instance, can release harmful byproducts into the atmosphere. These factors highlight the need for alternative synthesis methods that are both efficient and environmentally friendly [35].

Green synthesis approaches have gained attention in recent years as sustainable alternatives to traditional methods. The eco-friendly approaches prioritize lower energy consumption, non-toxic reagents, and minimal environmental impact while maintaining the effectiveness of oxide layer formation [29,32,36,37,38]. Pulsed laser synthesis is an example of an eco-friendly method for metal oxide production. Key advantages include the generation of nanomaterials without hazardous chemical reagents, process simplicity, and cost-effectiveness. Potential drawbacks may involve multi-step procedures for film deposition onto desired substrates, or high-energy requests [39,40,41,42]. Other studies have demonstrated the feasibility of CuO production using plant extracts, bacterial synthesis or fungal digestion [5,42,43]. Such biological methods not only eliminate the need for hazardous chemicals but also enable fine-tuning of oxide properties by leveraging natural biogenic agents [35,44].

In addition to biological synthesis, alternative chemical methods using alkaline environments have been explored. Studies indicate that exposing copper to the basic solution at moderate temperatures can facilitate CuO layer formation with minimal energy input [32]. The combination of heat and alkaline conditions enables controlled oxidation, ensuring a uniform and stable CuO layer. This approach significantly reduces the environmental footprint of traditional oxidation methods that rely on extreme heat or aggressive chemical agents.

To address these concerns, the present study aims to develop an eco-friendly approach for forming a tenorite (CuO) layer on a copper substrate. The objective is to implement a simple, rapid, and cost-effective procedure that utilizes relatively low temperatures and alkaline environments, thereby reducing the ecological footprint of CuO production while ensuring the structural integrity of the oxidized film. This sustainable approach not only minimizes the environmental risks associated with conventional methods but also promotes energy efficiency and operational safety in copper oxide film synthesis.

By leveraging a combination of immersion and vapour-phase exposure to alkaline solutions, this study seeks to optimize the oxidation process while maintaining a low processing temperature. The effectiveness of different treatment durations and temperatures is assessed to determine the optimal conditions for CuO layer formation. Unlike conventional high-temperature oxidation techniques, which often introduce internal stress and structural defects, this method preserves the mechanical stability of the copper substrate while ensuring a uniform and well-adhered oxide layer.

The oxide layer obtained on the copper surface was characterized using optical microscopy (OM), scanning electron microscope (SEM), peeling test, spectrocolorimetry, micro-Raman spectroscopy (µ-Raman) and X-Ray photoelectron spectroscopy (XPS) [45]. These analyses provide comprehensive insights into the structural, optical, and compositional properties of the CuO layer, confirming its successful formation through an environmentally sustainable approach [42,43].

This research underscores the growing necessity of green chemistry in materials science, emphasizing the importance of low-toxicity and energy-efficient approaches to oxide formation. By implementing sustainable methodologies, this study contributes to the development of advanced copper oxide coatings while minimizing environmental impact.

## 2. Materials and Methods

Based on Zhang et al. [32] and Neupane et al. [46], research was conducted to avoid a low range of temperatures in combination with NaOH solution to induce the formation of an oxide layer on the surface of copper. The oxide layer was induced using a 10% NaOH (*w*/*v*) solution. NaOH was purchased from Sodax (Sodax, Italy) and solubilized in distilled water.

Before the oxidation treatment, samples of pure copper 99.99% size 30 × 10 × 0.5 mm, were polished with 600, 800 and 1200 SiC paper, degreased with acetone, rinsed with ethanol, washed with distilled water and left to dry at room temperature.

A basic attack for copper oxide formation was carried out using the following methods (Table 1) based on the evidence reported in the literature [47,48,49].

The experimentation procedure and thermal oxidation were conducted in a closed glass vessel put into a silica oil bath. Silica oil was heated on an IKA RCT basic hotplate, and the temperature was controlled using an IKA ETS-D5 thermometer (IKA-Werke GmbH, Staufen im Breisgau, Germany).

Optical surface observation was performed using stereomicroscope Leica M125C acquired with DMC4500 USB (Leica, Wetzlar, Germany) digital camera and scanning electron microscopic (SEM) conducted using SEM Tescam-VEGA 3 (Microcontrolnt, Milan, Italy) at higher magnifications to investigate the morphology and homogeneity of the oxide layer. SEM analyses were performed operating with an accelerating voltage beam at 30 kV under a high vacuum at 12 mm working distance (WD). Imaging was performed in secondary electrons (SE) mode. Spectrocolorimetric analysis was conducted using a Y3060 3nh spectrophotometer (3nh, Guangzhou, China) equipped with an 8 mm aperture lens, enabling acquisition in SCE (Specular Component Excluded) mode. Spectra were collected in the visible region (400–700 nm), under D65 CIE Standard illuminant, with a Standard observer at a 10° angle. The colour was defined using CIELAB colour space coordinates. CIELAB parameters consist in the expression of lightness (L*) limited between 0 and 100 values, where zero is no colour and 100 corresponds to maximum brightness; colour variations from red to green (a*) and colour variations between blue and yellow (b*). An average of ten measurements were taken for each surface sample.

To determine the molecular structure of copper oxides, micro-Raman analysis was performed with a Renishaw RM 2000 Raman spectrometer (Renishaw, Pianezza, Italy), equipped with a CCD detector. The measurements were conducted in backscattering geometry. The Raman spectrometer is based on the Leica optical microscope. The objective has 50× magnification with 0.75 numerical aperture. The excitation of Raman scattering was performed with the 785 nm wavelength diode laser. The laser power was 1.58 mW at the sample. Spectra were collected with 1 s accumulation and 10 repetitions. Normalization 0 to 1 was conducted using Origin 8.5 software, and it was conducted on the values of the spectra collected to compare the intensities of the different spectra acquired on samples. Oxide layer cohesion to the metallic substrate was measured using the peeling test. The test was performed using an Instron 3343 dynamometer, (Instron, Italy) with a fixed angle of 90° and a controlled speed of 10 mm/min. The analysis was repeated on ten different specimens, and for each, the detachment force was recorded along a length of 50 mm. The Maximum Peel Value (MPV) was calculated as the highest force value recorded during the test, while the mean and standard deviation were determined to evaluate the reproducibility of the method [50,51].

XPS analysis was carried out by an ESCALAB 250Xi (Thermo Fisher Scientific Ltd., East Grinstead, UK), equipped with a monochromatic X-ray source (Al Kα—hν = 1486.6 eV) and 6-channeltron detection device. The measurements were performed operating in a UHV system at a base pressure of 1 × 10^−10^ mbar and using a constant pass energy of 50 eV. The samples were fixed to the sample holder by a metallic clip, in order to ensure the electrical contact, while the binding energy (BE) scale was calibrated positioning the Fermi level at BE = 0 eV. All spectra were collected and processed by Avantage software v5.9 (Thermo Fisher Scientific Ltd., East Grinstead, UK).

## 3. Results

Reported results investigate the impact of alkaline treatment using 10% NaOH solution and the role of temperature on the chemical and physical transformations of copper surfaces. The results provide insights into the morphological and chemical characteristics of the surface, to highlight the effectiveness of different methods in achieving desired outcomes.

**Figure 1:**
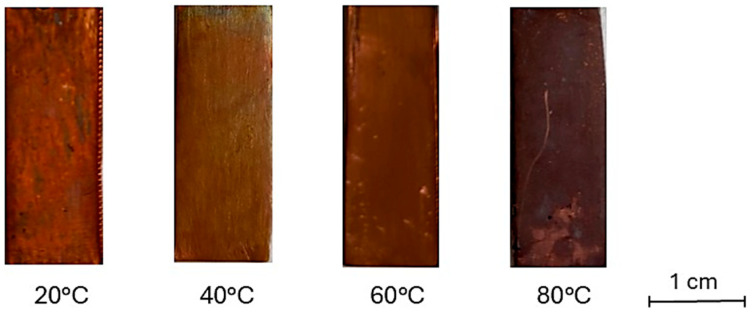
Surface hue of samples treated by immersion in NaOH 10% solution heated at different temperatures.

**Figure 2:**
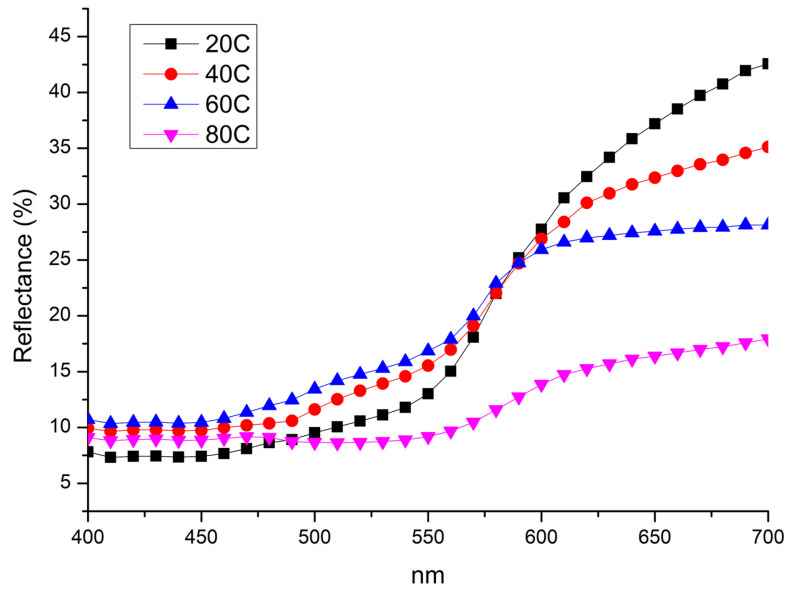
Reflectance spectra collected on samples treated by immersion in NaOH 10% at 20 °C (20C); 40 °C (40C); 60 °C (60C); and 80 °C (80C).

Figure 1 and Figure 2 show macroscopic images and reflectance spectra, respectively, of samples treated using method A (Im). Reflectance spectra (Figure 2) of samples treated at 20 °C and 40 °C exhibit a dominant wavelength at 650 nm. However, samples treated at 60 °C and 80 °C, corresponding to the surface changes observed in Figure 1, show a decrease in overall reflectance with increasing temperature and a sharp decrease in reflectance near 570 nm. The pure bulk Cu_2_O has is band gap of about 2.1 eV. It corresponds to about 570 nm wavelength. The defects as well as strains may shift the absorption edge value. Starting from this value, a sharp decrease in reflectivity begins to occur. This latter feature is due to the increased presence of oxides on the surface [52]. The enhanced formation of oxides leads to a darkening of the surface. Specifically, the sample immersed in the solution heated at 20 °C possesses an orange-brown hue similar to the elemental copper and, according to reflectance analysis (Figure 2), this sample shows the lightest hue among other samples. The presence of black areas on the surface indicates the growth of an inhomogeneous oxide layer. Samples immersed in solutions heated at 40 and 60 °C develop a more homogeneous oxidation layer while retaining an orange-brown hue. However, they appear darker compared to the sample treated at 20 °C. The sample immersed in the solution heated at 80 °C shows a dark, purplish-brown surface. The hue observed in samples treated at 20, 40 and 60 °C is characterized by varying degrees of orange-red-brown hue, suggesting the formation of a cuprite layer [53] on the surface accompanied by tenorite or paramelaconite, as indicated by a decrease in reflectance. The sample treated by immersion at 80 °C was prevalently characterized by a darkish appearance, suggesting an increasing possibility of tenorite formation.

**Figure 3:**
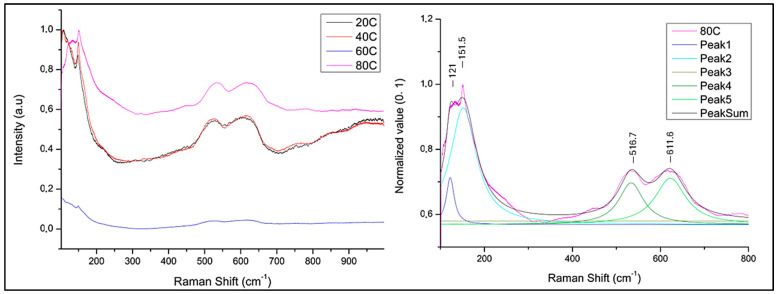
µ-Raman spectrum of the sample treated by immersion in NaOH 10% solution heated at different temperatures (**left**) and deconvolution of 80 °C treated sample (**right**).

µ-Raman analyses were performed on extended darker areas on the sample surface to investigate the nature of Cu-oxide film (Figure 1 and Figure 2). All compared spectra were normalized from 0 to 1 (minimum–maximum intensity value normalization). Spectra acquired on sample obtained at 80 °C (Figure 3) is characterized by peaks at 151, 516, and 611 cm^−1^. Peaks at 151 and 516 cm^−1^ are attributable to Cu_2_O [54,55] while at 611 cm^−1^ present in the sample spectrum can be traced back to the Cu_2_O peak positioned at 634 cm^−1^. Shift from 634 to 611 cm^−1^ associated with the enlarged peak shape suggests a poor crystallization of the Cu_2_O phase [56,57].

**Figure 4:**
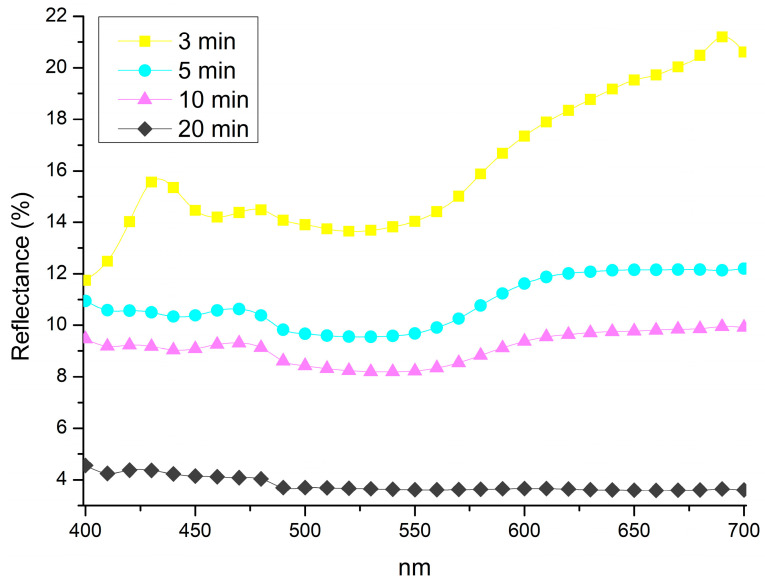
Spectra collected from samples treated by 1 min immersion and exposed to vapour for 3 min (3 min); 5 min (5 min); 10 min (10 min); and 20 min (20 min).

The results obtained using method B are presented below. The reflectance spectra (Figure 4) indicate that longer exposure times promote the formation of a darker patina. The spectrum recorded at 3 min exhibits spectral features at 425, 475, and 650 nm. At 5 and 10 min, the spectra display an increasingly linear trend, which becomes more pronounced at 20 min, where the surface develops a nearly colourless appearance, ultimately resulting in a deep black coloration.

**Figure 5:**
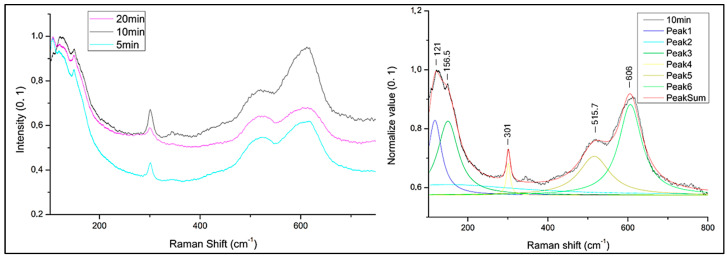
Comparison of µ-Raman spectra collected by sample treated with 1 min immersion and different exposition time to solution vapours: 5 min exposition to vapour (5 min); 10 min exposition to vapour (10 min) and 20 min exposition to vapour (20 min) (**left**); Deconvolution of µ-Raman spectra collected from the sample (10 min) treated with 1 min immersion and 10 min exposition to vapour solution, heated at 80 °C and magnetically stirred (**right**).

To define the composition of darker oxide, µ-Raman spectra were collected from treated samples with method B at 5, 10 and 20 vapour exposition time. Samples exposed to vapour for 3 min were excluded due to the absence of blackish hue and relative tenorite formation, as confirmed by µ-Raman and colorimetric results obtained from method A. All spectra from the selected treated samples with method B are characterized by the presence of similar main peaks (Figure 5), although the ratio of peak intensities is different in relationship to the exposition time. The main peaks are at 121, 156, 301, 515, and 606 cm^−1^. The highest intensity of the peaks at 301 and 606 cm^−1^ is assigned to the sample treated for 1 min of immersion and 10 min exposure to NaOH 10% solution. Peaks at 301 cm^−1^ are due to a shift in the main characteristic CuO peaks at 297–298 cm^−1^ [32,36,58]. The peak at 515 cm^−1^ is attributable to the intrinsic violation mechanism of selection rules in Cu_2_O phonon frequencies during structure orientation passage into paramelaconite Cu_4_O_3_ [59], but it is also attributed to CuO [60,61]. Support for attributing this peak to paramelaconite is reported in the study of Jagadish et al. [62] where the peak at 532 cm^−1^ is caused by Raman-allowed paramelaconite crystals’ mode. The peak at 606 cm^−1^ can be attributed to a Cu-O stretching mode [59] shifted from 602 cm^−1^, and similar to the previous samples. The variation in the shape of the latter peak indicates changes in the crystal structure induced by the temperature dependence of formation [32,60,61,62].

To investigate whether the formation of the copper oxide layer by exposure to the vapours of NaOH solution also occurs in the absence of immersion of the sample for 1 min in the same solution, an analysis was carried out by exposing the sample only to the vapours, without prior immersion. Reflectance spectra were collected, followed by optical microscopy (OM) imaging to assess the surface appearance. Finally, µ-Raman spectra were acquired from three different samples: one exposed exclusively to vapours (Vap), one subjected to immersion followed by 10 min of vapour exposure (10 min), and one treated solely by immersion in the 80 °C NaOH solution (80C).

**Figure 6:**
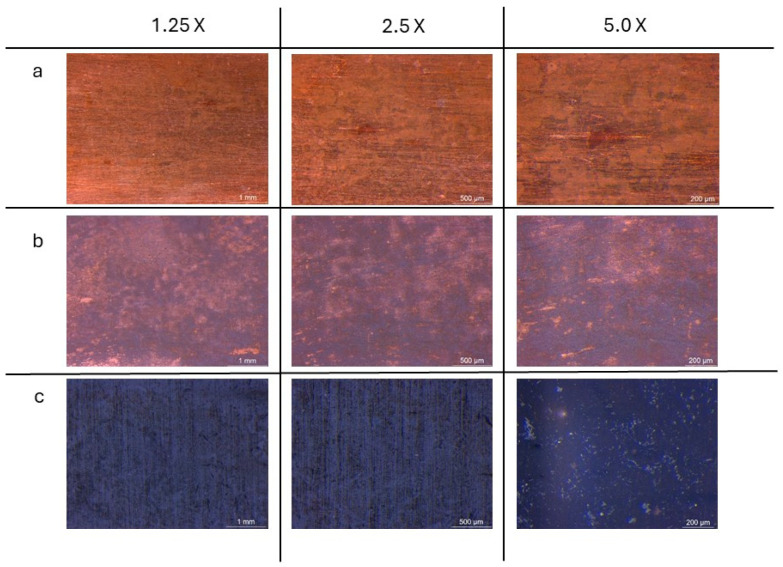
OM images of surface samples from samples treated with vapour exposition treatment (**a**); immersion treatment (**b**) and combining immersion and vapour exposition treatment (**c**), observed at 1.25×, 2.5× and 5.0× magnification.

Images collected through OM observations (Figure 6) reveal distinct oxidation patterns resulting from the three tested methods. The sample exposed exclusively to NaOH vapours exhibits an orange-brown coloration with non-homogeneous red oxidation spots. More pronounced oxidation is observed in the samples treated by immersion, which display a combination of orange-brown regions interspersed with larger blackened areas, indicating a more advanced oxidation process. The most uniform and intense oxidation is found in the sample subjected to both immersion and subsequent vapour exposure, where the surface is entirely covered by a dark-coloured patina, suggesting the formation of a more continuous and well-developed oxide layer.

**Figure 7:**
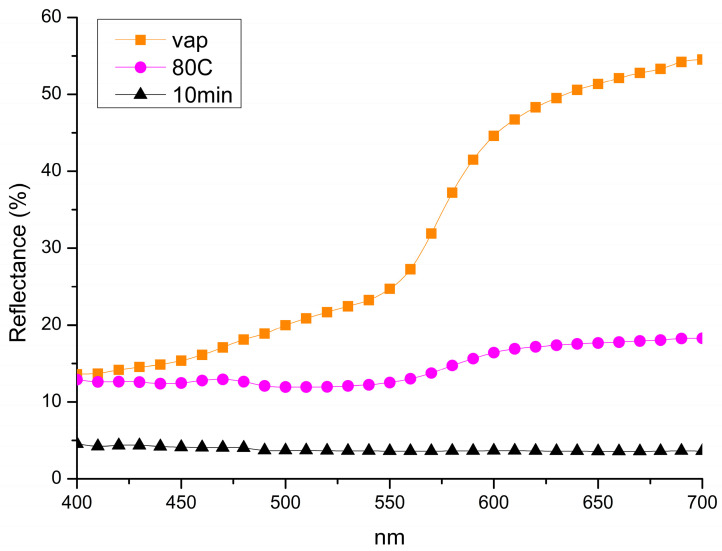
Comparison of reflectance data collected from samples treated only by vapours exposition (Vap), samples treated only by immersion treatment (80C) and samples treated by combining immersion and exposition treatments (10 min).

A comparison of the reflectance spectra analysis of the samples corroborates the OM observations, indicating a higher reflectance percentage and lighter coloration in the sample exposed exclusively to NaOH vapours. In contrast, samples treated by only immersion or by the combined immersion–vapour process exhibit lower reflectance values, corresponding to a darker surface appearance. Among these, the sample subjected to both immersion and vapour exposure presents the lowest reflectance and the most intense dark coloration, suggesting a more advanced oxidation process and a higher concentration of CuO (Figure 7). Table 2 presents the CIE L*a*b* colour coordinate values for samples treated with the three methods. According to OM analysis and reflectance spectra, samples exposed only to vapours exhibited the highest lightness (L*) and yellowness (b*), indicating minimal surface alteration (i.e., oxide formation). Increasing the treatment temperature and combining the methodologies, all the colours shift toward a darker and cooler hue.

**Figure 8:**
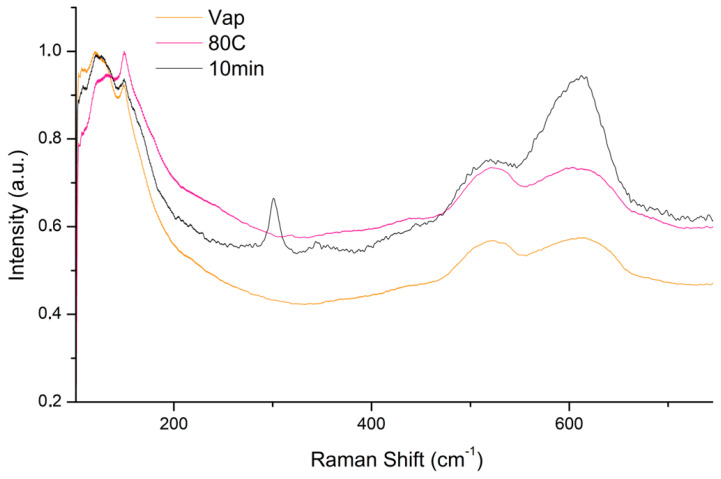
Comparison of micro-Raman spectra collected by sample treated with vapour exposition (Vap), immersion in solution heated at 80 °C (80C) and 1 min immersion and 10 min vapour exposition (10 min) of 10% NaOH solution.

µ-Raman spectra collected on the three selected samples Vap, 80C and 10 min (Figure 8), show a peak attributed to tenorite (301, 515 and 606 cm^−1^), only in the sample treated by immersion and subsequent exposure to the vapours of the 10% NaOH solution heated to 80C (method B). In the spectra collected on the other two samples, only the peaks attributed to cuprite (121, 151, 516 and 606 cm^−1^) are present [55].

XPS studies were performed to identify the surface chemical composition of the sample, including the oxidation state of copper on the three different treatment methods, and to confirm the formation of CuO on the surface of the sample. The obtained results are listed in Table 3, Table 4 and Table 5 where it can be seen that all the samples were characterized by the presence of Cu, C, O and, with the exception of the one exposed to vapour of NaOH. The peak-fitting analysis evidence that C 1 s signal included several contributions found at BE = 285.0, 286.4 eV and 288.4 eV assigned to C–C bond, C–O or C = O bond and carboxylic groups, respectively. In the case of the sample immersed in the NaOH solution, the C 1 s signal also showed the contribution of the carbonate, at BE = 289.1 eV [62].

**Figure 9:**
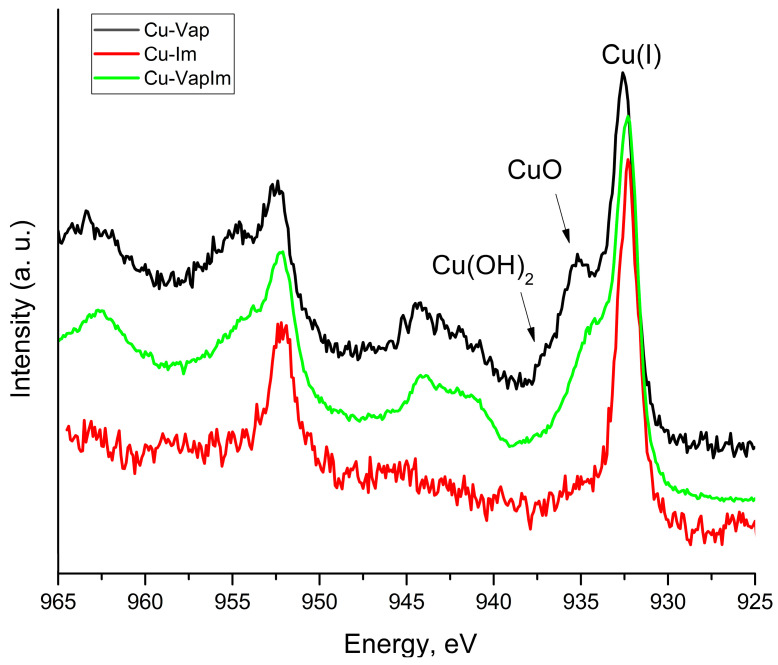
80C (Cu-Im), Vap (Cu-Vap) and 10 min (Cu-VapIm) XPS spectra.

As it concerns the Cu 2p signal, Figure 9 shows that the shape of the signal changes depending on the corrosive treatment. When the sample was immersed in the NaOH solution, a single peak, positioned at BE = 932.2 eV, was found. This value could be ambiguous because it is characteristic of both metallic and Cu^+1^. Auger parameter can be used to discern this issue. This parameter is calculated by applying the following Formula (4):

α′ = BE (Cu 2p3/2) + KE (Cu LMM)(4)

where BE (Cu 2p3/2) is the BE of the core level Cu 2p3/2, while KE (Cu LMM) is the kinetic energy of the photo-induced Auger transition. The obtained value α′ = 1849.1 ÷ 1849.6 eV suggested that Cu was oxidized to +1, rather than being metallic. However, in the other samples, an adjunctive component was found, as a consequence of these treatments, at BE = 934.2 eV and 935.6 eV, was assigned to CuO and Cu(OH)_2_, respectively, characterized also by their typical satellite peaks located at higher BE (Figure 9) [63].

**Figure 10:**
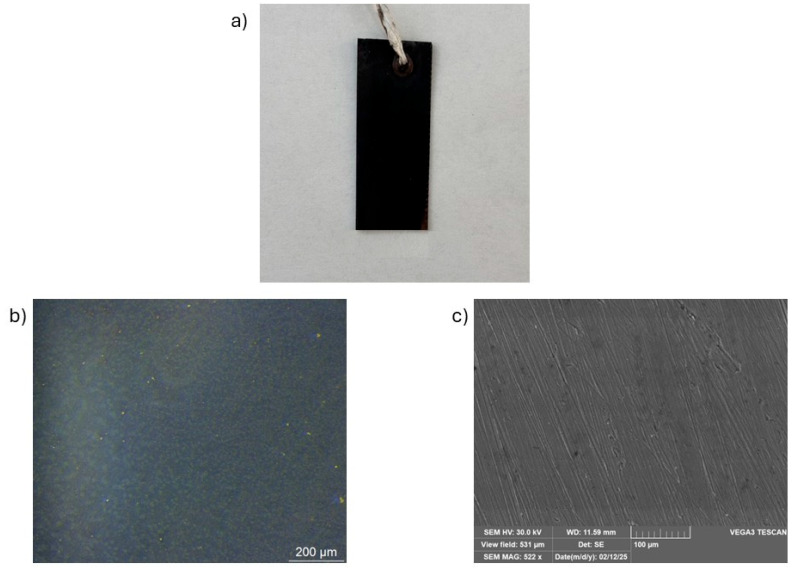
Observation of (**a**) oxide layer on general images, (**b**) optical microscope 5×, and (**c**) SEM magnification 10 µm resolution.

Scanning electron microscopy (SEM) (Figure 10) and peeling test (Table 6) were conducted exclusively on samples obtained using method B, as this approach yielded the most promising results in terms of oxide layer uniformity, morphology, and adhesion to the substrate.

The images acquired through secondary electron (SEM-SE), with 10 µm resolution, reveal a highly homogeneous and uniform oxide layer on the sample surface (Figure 10c). The presence of scratches, due to the initial polishing of the sample surface with SiC paper, still remains visible.

SEM, which provides higher-resolution topographical information, confirms the formation of a continuous and homogeneous CuO layer, indicating that the inhomogeneities observed by OM are likely due to optical magnification rather than actual surface irregularities.

They are widely used due to their simplicity and speed, especially in characterizing thin films and determining the adhesion strength of materials [50,64]. In Table 6, the peeling test data for three samples prepared using method B are reported. The results indicate a moderate adhesion between the oxide layer and the copper substrate, with Maximum Peel Values (MPV) ranging from 0.0436 N/mm to 0.0523 N/mm. While these values suggest a relatively consistent adhesion across the tested samples, they remain lower than those typically reported for CuO films deposited via methods such as PVD or CVD.

## 4. Discussion

The aim of this study was to develop a more environmentally friendly approach for the formation of a tenorite (CuO) layer on a metallic copper substrate. The proposed method prioritizes sustainability while also being simple, fast, non-toxic, and cost-effective, ensuring both eco-compatibility and time efficiency.

By leveraging the known effectiveness of thermal treatment and exposure to alkaline environments, three different procedures were tested to enhance cost efficiency, reduce environmental impact and procedural simplicity. The first method involved immersing the copper sample in a heated alkaline NaOH solution at varying temperatures. Temperature increments of 20 °C were applied within the range of boiling water temperatures to assess the role of heat in oxide formation. Optical microscopy and spectrocolorimetric analysis confirmed that immersion in a basic solution, combined with thermal treatment (heating range 20–80 °C), promotes the development of a dark oxide layer on the copper surface. A comparison of data collected at identical immersion time (30 min) revealed that only the highest temperature tested resulted in the formation of a visibly dark oxidation layer. Tenorite, characterized by its bluish-grey-black appearance, was expected in these conditions. µ-Raman analysis did not detect tenorite on the immersion-treated sample, instead indicating the presence of cuprite (Cu_2_O). These results suggest that a temperature close to 100 °C is necessary but not solely sufficient to catalyze the formation of tenorite on the copper surface. To further enhance oxide formation, the second experimental approach combined immersion in a heated 10% NaOH solution with subsequent exposure to its vapours for varying durations. This combined treatment yielded promising results. Both reflectance analysis and microscopic observations indicated the formation of a dark-coloured oxide layer consistent with tenorite. µ-Raman analysis, however, revealed the presence of peaks associated with both paramelaconite (Cu_4_O_3_) and tenorite. Paramelaconite appears to be an intermediate crystalline phase between cuprite and tenorite, as suggested by overlapping spectral features. Specifically, the presence of a peak around 298–300 cm⁻^1^ suggests a structural relationship between paramelaconite and tenorite, with the former resembling CuO but lacking monoclinic distortion and exhibiting partial oxygen charge removal [25,65]. Previous studies indicate that annealing treatments can induce the transformation of paramelaconite into tenorite by stabilizing its crystalline phase [62]. A third treatment was conducted as a comparative parameter, where the copper sample was exposed only to the vapours of a 10% NaOH solution heated to 80 °C under magnetic stirring. This treatment, performed at relatively low temperatures and for a short duration, proved insufficient for inducing tenorite formation on the copper surface without additional processing. The obtained oxide layer with method B, identified as the most promising procedure to induce the formation of tenorite on the copper surface, was examined by SEM to assess its homogeneity and morphology and by peeling test to assess the adhesion between the formed oxide layer and substrate. The obtained results indicate that this method yields an excellent outcome in terms of uniformity, morphology, and adhesive characteristics of the oxide layer.

Compared to CuO films obtained through wet chemical synthesis or sol–gel methods, which typically exhibit adhesion values below 0.1 N/mm due to weaker interfacial bonding and higher porosity, the present results demonstrate comparable performance.

However, the adhesion of the CuO films produced in this study remains lower than that generally observed for films deposited via physical vapour deposition (PVD) or chemical vapour deposition (CVD) [66,67].

Although adhesion values for PVD- and CVD-grown CuO films are reported to be higher, precise numerical values vary significantly depending on deposition parameters such as temperature, pressure, and substrate preparation. Some studies indicate that optimized deposition conditions can lead to strong film–substrate bonding, particularly when high-energy deposition processes promote interfacial diffusion and chemical bonding.

This suggests that while the proposed low-temperature oxidation method offers an energy-efficient and environmentally friendly alternative, further optimization may be necessary to enhance film adhesion. Strategies such as post-synthesis annealing or surface pretreatment of the copper substrate, both of which have been shown to significantly improve adhesion in oxide coatings, could be explored to strengthen the interfacial bonding between the oxide layer and the substrate. Furthermore, this study demonstrates that modifying alkaline treatment parameters, such as a combination of methodology, extending exposure time to NaOH vapours or incorporating surfactants to enhance surface wetting, can yield a homogeneous and mechanically robust oxide film even at lower temperatures, thereby achieving comparable outcomes to those obtained with more conventional methods.

X-ray photoelectron spectroscopy (XPS) analysis confirmed that the immersion-plus-vapour treatment successfully induced the formation of a thin CuO layer. However, the detection of additional chemical compounds suggests that the process requires refinement. These secondary compounds likely contributed to the spectral shifts observed in µ-Raman analysis and the imperfect black coloration of the sample surface. It is reasonable to infer that their presence interfered with the formation of a well-defined crystalline CuO phase. Further optimization of the treatment parameters, such as exposure time and temperature control, may enhance the purity and crystallinity of the resulting tenorite layer.

## 5. Conclusions

The three methods selected to induce the formation of tenorite on a metallic copper substrate reflect the most used treatments for the formation of oxides on metal surfaces. The challenge brought by this study was to exploit these treatments by using them to induce the formation of a tenorite layer on the surface of metallic copper using low-cost, environmentally safe, low-temperature, and time-saving experimental conditions. The results demonstrate how this study was successful in achieving this requirement. Even using temperatures below 100 °C in varying the parameters of the basic environment and a combination of immersion treatment and vapour exposure, it is possible to obtain a CuO layer in a brief time and with low costs. Further research on the uniformity and adherence of the produced CuO layer may be carried out.

## Figures and Tables

**Table 1:** Description of the selected treatment method, related to the sample code.

Sample Code	Method Description
Im	Method A: immersing copper samples for 30 min in a 10% NaOH solution, heated at different temperatures, respectively, 20, 40, 60, and 80 °C. Solutions were magnetically stirred at 500 rpm.
ImVap	Method B: immersing the sample for 1 min in a 10% NaOH solution, which was stirred at 500 rpm and heated to 80 °C, followed by exposure to the vapour of the same solution at different times of exposition: 3, 5, 10 and 20 min.
Vap	Method C: by contact with a strongly basic solution (pH 14), involved treating the sample by exposure only to vapours of 10% NaOH solution, magnetically stirred at 500 rpm and heated to 80 °C. Exposure time to the vapour was conducted for 5, 10 and 20 min.

**Table 2:** Colour space CIELAB values obtained on samples treated with the three methods.

Method	Sample Code	L*	a*	b*
A: Im	20 °C	48.1	22.1	25.1
	40 °C	50.2	16.0	20.0
	60 °C	49.5	10.9	18.5
	80 °C	38.8	10.1	4.9
B: ImVap	3 min	45.7	6.3	1.3
	5 min	38.4	4.5	−0.2
	10 min	35.4	3.2	−1.4
	20 min	22.5	0.9	−2.7
C: Vap	Vap	52.3	21.2	28.3

**Table 3:** Cu-Vap (Vap) XPS sample results.

Name	Peak BE (eV)	FWHM (eV)	Area (P) CPS.eV	Atomic %	Assignment	Auger Parameter α′ (eV)
C1s-1	285.0	1.69	17,108.94	61.8	C–C	
C1s-2	286.4	1.69	1448.38	5.2	C–O, C = O	
C1s-3	288.4	1.69	1397.89	5.1	-COOH(R)	
Cu2p3-1	932.5	1.68	15,757.20	4.4	Cu(I)	1849.1
Cu2p3-2	935.2	2.39	8147.80	2.3	Cu(OH)_2_	
O1s-1	531.9	1.88	8969.87	11.8	hydroxides	
O1s-2	530.5	1.88	5539.83	7.3	Oxides	
O1s-3	533.5	1.88	1610.63	2.1	H_2_O	

**Table 4:** Cu-Im (80C) XPS sample results.

Name	Peak BE (eV)	FWHM (eV)	Area (P) CPS.eV	Atomic %	Assignment	Auger Parameter α′ (eV)
C1s-1	285.0	1.57	20,268.04	33.4	C–C	
C1s-2	289.1	1.57	6638.69	10.9	Carbonates	
Cu2p3	932.2	1.56	11,748.05	1.5	Cu(I)	1849.2
Na1s	1071.6	1.84	64,061.97	18.0	Carbonates	
O1s	531.3	1.86	47,907.67	28.8	Carbonates	

**Table 5:** Cu-ImVap (10 min) XPS sample results.

Name	Peak BE (eV)	FWHM (eV)	Area (P) CPS.eV	Atomic %	Assignment	Auger Parameter α′ (eV)
C1s-1	284.8	1.53	15,548.59	39.4	C–C	
C1s-2	286.5	1.53	2145.08	5.4	C–O, C = O	
C1s-3	288.4	1.53	1419.61	3.6	-COOH(R)	
Cu2p3-1	932.3	1.51	52,744.65	10.3	Cu(I)	1849.6
Cu2p3-3	935.6	2.72	9783.49	1.9	Cu(OH)_2_	
Cu2p3-2	934.2	2.06	23,691.50	4.6	CuO	
Na1s	1071.5	1.77	4462.76	1.9	Carbonates	
O1s-1	530.0	1.56	19,970.00	18.4	Oxides	
O1s-2	531.5	1.56	11,611.16	10.7	Hydroxides	
O1s-3	533.1	1.56	3936.54	3.6	H_2_O	

**Table 6:** Peeling test.

Samples	Maximum Peel Value	Standard Deviation
1	0.0523	0
2	0.0436	9.43 × 10^−0.5^
3	0.0457	2.36 × 10^−0.5^
4	0.0578	2.36 × 10^−0.5^
5	0.0449	4.71 × 10^−0.5^
6	0.0493	2.36 × 10^−0.5^
7	0.0513	0
8	0.0445	4.71 × 10^−0.5^
9	0.0519	2.36 × 10^−0.5^
10	0.0439	2.36 × 10^−0.5^

## Data Availability

The original contributions presented in the study are included in the article. Further inquiries can be directed to the corresponding author.
